# Next-generation sequencing for DLBCL patients with early failure after frontline R-CHOP chemo-immunotherapy

**DOI:** 10.3389/fonc.2026.1682952

**Published:** 2026-02-18

**Authors:** Liu Shi, Xiaohua Liu, Jing Wang, Yong Su, Xiaochang Gong, Di Deng

**Affiliations:** 1 Department of Radiation Oncology, Jiangxi Cancer Hospital of Nanchang Medical College, Nanchang, China; 2 National Health Commission (NHC) Key Laboratory of Personalized Diagnosis and Treatment of Nasopharyngeal Carcinoma (Jiangxi Cancer Hospital of Nanchang Medical College), Nanchang, China; 3 Department of Esophageal, Mediastinal and Lymphatic Oncology, Zhongnan Hospital, Wuhan University, Wuhan, Hubei, China; 4 Hubei Key Laboratory of Tumor Biological Behaviors, Wuhan, Hubei, China; 5 Cancer Clinical Study Center, Zhongnan Hospital of Wuhan University, Wuhan, Hubei, China

**Keywords:** diffuse large B-cell lymphoma, DLBCL, early failure, interim treatment responses, next-generation sequencing

## Abstract

**Background:**

Early failure less than 12 months (POD12) of frontline R-CHOP chemo-immunotherapy in very poor outcomes and requires alternative therapy in patients with Diffuse large B-cell lymphoma (DLBCL).

**Aim:**

We aim to evaluate the association of gene alterations and clinical factors with POD12.

**Methods:**

The panel included 103 genes that were examined in 26 patients with newly diagnosed DLBCL treated with standard R-CHOP chemo-immunotherapy in the frontline setting therapy using next-generation sequencing. The association of clinical features and gene alterations with early progression was analyzed.

**Results:**

POD12 group (n=12) was related to poorer OS with a hazard ratio (HR) of 12.13 (95% confidence interval [CI] 2.34–62.78, p=0.0029). Genes mutated in 96.15% of patients (25/26) were grouped into 11 specific pathways, and the POD12 subtype was mostly characterized by mutations in the epigenetic modulation pathway (33.32% of total variation) and apoptosis/cell cycle/autophagy pathway (20.83% of total variation), whereas the no-POD12 subtype is mostly characterized by mutations in the epigenetic modulation pathway (40.66% of total variation). CD79B mutation frequency was significantly increased in the no-POD12 group compared with the POD12 group (50.00% vs 8.33%, p=0.0357). Not achieving complete response (CR) during interim treatment response was found to be significantly associated with the occurrence of POD12 (p=0.0214).

**Conclusions:**

CD79B wide-type and not achieving CR during interim responses evaluation correlate with POD12. These findings provide a basis for the development of optimal alternative therapies in clinical trials.

## Introduction

Diffuse large B-cell lymphoma (DLBCL) is the most common form of adult lymphoma worldwide, accounting for 30–40% of newly diagnosed Non-Hodgkin Lymphoma (NHL) (1). It is a clinically aggressive and heterogeneous disease with established diversities in clinical outcomes, genomic characteristics, and cell of origin (COO). Standard DLBCL therapy remains rituximab, cyclophosphamide, doxorubicin, vincristine, and prednisone (R-CHOP) chemo-immunotherapy (2). Over 60% of patients can achieve a complete cure or yield long-term survival with first-line R-CHOP treatment (3). The rest, however, eventually succumbed to recurrent or refractory disease. In particular, patients who with early failure of frontline chemo-immunotherapy within less than 12 months (POD12) often experience very poor outcomes and commonly did not benefit from salvage therapy even in combination with autologous stem cell transplantation (4). Consequently, it is necessary to provide them with a more intense treatment beyond standard R-CHOP treatment in the setting of frontline therapy. It is important to predict high-risk patients for POD12 in newly diagnosed DLBCL patients. The International Prognostic Index (IPI) is the most commonly used prognostic index to predict the prognosis of DLBCL patients. Prognostic evaluation and risk stratification were performed by IPI based on 5 aspects, including age, Ann Arbor stage, lactate dehydrogenase (LDH) levels, physical condition score, and the number of extranodal organ involvement. However, DLBCL patients with the same IPI score might still have different outcomes after receiving similar chemotherapy due to tumor heterogeneity (5).

Next-generation sequencing (NGS) technologies, enabling high-throughput DNA sequencing, have emerged over the past decade, and have provided new insights into the genomic characterization of DLBCL by identifying recurrent single nucleotide variants (SNV) and insertion-deletion events (INDEL) (6, 7). Recently, many gene alterations have been identified using whole-exome and transcriptome sequencing in DLBCL samples (8–11). Many of these somatic mutation target genes play a crucial role in B- cell function (BCR signaling, NF-kB pathway, NOTCH signaling), JACK-STAT, MAP-kinases, immunity, cell cycle/apoptosis, or epigenetic regulation (12). Importantly, certain recurrent SNVs are potentially actionable mutations, highlighting the importance of targeted therapy development.

In this study, we analyzed a panel of 103 genes in a cohort of patients with newly diagnosed DLBCL treated with standard R-CHOP chemo-immunotherapy. We aim to evaluate the association of clinical factors and gene alterations with POD12.

## Methods

### Patients

A total 26 patients with newly diagnosed DLBCL who were treated with standard R-CHOP chemo-immunotherapy in the frontline setting used in this study were collected from the Zhongnan Hospital of Wuhan University. Clinical characteristics and outcomes were extracted from the electronic medical record. For all patients, pathology was reviewed and confirmed at Zhongnan Hospital of Wuhan University. This study was reviewed and approved by the hospital’s Institutional Review Boards with the informed consent of the patients.

### Immunohistochemistry

3-μm paraffin sections were immunohistochemically analyzed by the indirect immunoperoxidase method. Anti-CD10 (Abcam, EPR22867-118), BCL6 (Abcam, EPR11410-43), MUM1 (Abcam, ab247079), BCL2 (Abcam, EPR17509), and C-MYC (Abcam, EPR17924) antibodies were used. The COO subgroups were determined by Han’s classification.

### Next-generation sequencing assays

Genomic DNA from tumor tissue and patient-matched normal blood was subjected to targeted Next-generation sequencing (NGS) assay. Briefly, genomic DNA was extracted from tumors and patient-matched blood samples to generate barcoded libraries. After the capture of exons and selected introns of the genes included in the sequencing panel using custom biotinylated DNA probes and streptavidin-conjugated beads, pooled libraries were sequenced on the Illumina HiSeq 2500 system. The panel included 103 genes. The resulting sequences were run through an optimized informatics pipeline to identify somatic alterations covering genes for sequence mutations comprising single nucleotide variants (SNVs) and insertion-deletion events (indels).

### DNA extraction and sequencing library preparation

Formalin-fixed, paraffin-embedded (FFPE) tumor sections were processed at a CLIA/CAP-accredited central laboratory (Shanghai Renwei Technology Inc., Shanghai, China) for targeted next-generation sequencing (NGS) based on hybrid capture, covering a panel of 103 cancer-associated genes.

Briefly, genomic DNA (gDNA) was isolated from 5 to 8 sections of 10 µm FFPE tissue. Deparaffinization was performed using xylene, followed by gDNA extraction with the QIAamp DNA FFPE Tissue Kit (Qiagen) according to the manufacturer’s protocol. DNA concentration was measured using a Qubit 3.0 Fluorometer (Thermo Fisher Scientific), and purity was assessed with a NanoDrop 2000 spectrophotometer (Thermo Fisher Scientific). Purified gDNA was fragmented to an average size of ~350 bp using a Covaris M220 ultrasonicator (Covaris), and size selection was performed with Agencourt AMPure XP beads (Beckman Coulter).

Sequencing libraries were constructed from the size-selected gDNA using the KAPA Super Library Preparation Kit (KAPA Biosystems) following the manufacturer’s guidelines. Libraries were amplified by PCR and purified with Agencourt AMPure XP beads prior to target enrichment.

For hybridization capture, 2 µg of each indexed library was combined with a custom panel of biotinylated probes (Integrated DNA Technologies) using IDT xGen Lockdown Reagents. Post-capture amplification was carried out with KAPA HiFi HotStart ReadyMix (KAPA Biosystems), followed by cleanup with Agencourt AMPure XP beads. The final libraries were quantified via qPCR using the KAPA Library Quantification Kit (KAPA Biosystems), and size distribution was evaluated on a Bioanalyzer 2100 (Agilent Technologies). Paired-end sequencing (2 × 150 bp) was performed on an Illumina HiSeq 2500 platform according to standard protocols.

### Sequence alignment and data processing

Base calling and demultiplexing were conducted using bcl2fastq v2.16.0.10 (Illumina), generating FASTQ files in Illumina 1.8+ encoding. Raw reads underwent quality control and adapter trimming with Trimmomatic. High-quality reads were aligned to the human reference genome (GRCh37/hg19) using BWA-MEM (BWA version 0.7.12) with default parameters. SAM files were converted to sorted and indexed BAM files using Picard tools (v1.119). Local realignment around indels and base quality score recalibration (BQSR) was performed with the Genome Analysis Toolkit (GATK, version 3.4-0).

### Variant detection and annotation

Single-nucleotide variants (SNVs) and short insertions/deletions (indels) were detected using VarScan2 (v2.3.9) with the following thresholds: minimum variant allele frequency = 0.01 and p-value < 0.05. Called variants were annotated with ANNOVAR and visually verified using the Integrative Genomics Viewer (IGV). Copy number variations (CNVs) were identified using an in-house developed algorithm.

### Biostatistics

Deidentified clinical information was extracted from the medical record. Interim treatment response scan for each patient after 4 cycles of R-CHOP chemo-immunotherapy was determined radiographically by independent evaluation by a radiologist based on the Lugano Response Criteria for Non-Hodgkin Lymphoma (13).

Associations between the different variables and clinical parameters were assessed by using the chi-square (and Fisher’s exact) test. Kaplan–Meier analyses were used to assess survival rates and log-rank tests were used to determine the statistical significance. Overall survival (OS) was calculated from the date of diagnosis to death from any cause or last follow-up. Progressive-free survival (PFS) was calculated from the date of diagnosis to the date of relapse or date of death from any cause, or the last follow-up date, whichever occurred first. p-values < 0.05 were considered significant. All statistical analyses were performed using the Statistical Package for the Social Sciences, v. 26.0 (IBM SPSS, USA).

## Results

### Clinical characteristics of the patients

Tissue specimens from 26 patients with newly diagnosed DLBCL treated with standard R-CHOP chemo-immunotherapy in our institution from Dec 1, 2018, to Jun 11, 2021, were collected, sequenced, and analyzed. The last follow-up date was Nov 1, 2021, and the median follow-up was 23 months (range 3–35 months). Demographic and clinical characteristics are shown in **Table 1**. The gender distribution was well balanced except more patients were female (53.8%) and more patients were younger than 60 years old (n=19, 73.1%). Sixteen patients were presented as advanced stage DLBCL (Ann Arbor stage III-IV in 61.5%). Fifteen patients had normal serum LDH levels. Fifteen patients had good physical condition (0–1 ECOG score of 57.7%). Only 3 patients had more than 1 extranodal site involved (11.5%). Seventeen patients presented a low-risk DLBCL (0–2 IPI score 0–2 in 65.4%). The majority of patients do not have B symptoms (n=20, 76.9%). Ten patients had tumors size larger than 5cm (38.5%). Using Hans’ algorithm, more non-GCB DLBCL were observed than GCB (53.8 versus 42.3%). Using Ki67 staining, more than half of the cases showed ≥80% proliferation index (n=17, 65.4%). Patients received a median of six courses (range, 1–6) of R-CHOP chemo-immunotherapy. Interim treatment responses were scored based on Lugano Response Criteria for Non-Hodgkin Lymphoma as follows: complete response, CR (n=11, 42.3%), no CR (n=15, 53.8%).

**Table 1:** Baseline characteristic of patients and interim treatment response.

Clinical characteristics	Overall (n=26)	POD12 (n=12)	no-POD12 (n=14)	p-value
Gender				0.716
Male	12 (46.2)	6 (50)	6 (42.9)	
Female	14 (53.8)	6 (50)	8 (57.7)	
Age, year				0.081
≤60	19 (73.1)	11 (91.7)	8 (57.7)	
>60	7 (26.9)	1 (8.3)	6 (42.9)	
Stage of disease				0.248
I-II	10 (38.5)	3 (25)	7 (50)	
III-IV	16 (61.5)	9 (75)	7 (50)	
Serum LDH level				0.126
Normal	15 (57.7)	5 (41.7)	10 (71.7)	
Elevated	11 (42.3)	7 (58.3)	4 (28.6)	
ECOG performance status				0.126
0-1	15 (57.7)	5 (41.7)	10 (71.7)	
≥2	11 (42.3)	7 (58.3)	4 (28.6)	
No. of extranodal sites involved				0.580
0-1	23 (88.5)	10 (83.3)	13 (92.9)	
≥2	3 (11.5)	2 (16.7)	1 (7.1)	
IPI risk group				0.683
0-2	17 (65.4)	7 (58.3)	10 (71.7)
3-5	9 (34.6)	5 (41.7)	4 (28.6)
B symptom				0.365
Absence	6 (23.1)	4 (33.3)	2 (14.3)
Presence	20 (76.9)	8 (66.7)	12 (85.7)
Tumor size, cm				0.422
<5	16 (61.5)	6 (50)	10 (71.7)
≥5	10 (38.5)	6 (50)	4 (28.6)
Cell of Origin (Hans)				0.233
GCB	11 (42.3)	7 (58.3)	4 (28.6)
Non-GCB	14 (53.8)	5 (41.7)	9 (64.3)
NA	1 (3.8)	0 (0)	1 (7.1)
Proliferation index (Ki67)				
≥80%	8 (30.8)	4 (33.3)	4 (28.6)	>0.999
<80%	17 (65.4)	8 (66.7)	9 (64.3)
NA	1 (3.8)	0 (0)	1 (7.1)
Interim treatment response				**0.014**
CR	11 (42.3)	2 (16.7)	9 (64.3)
no CR	15 (57.7)	10 (83.3)	5 (35.7)

***^a^*** Chi-square test or Fisher’ exact test. Data are n (%) unless otherwise indicated.

The bolded items indicate statistical significance, P <0.05.

### Impact of POD12 DLBCL on outcomes

The associations of gender, age, stage of disease, serum LDH level, ECOG performance status, the number of extranodal involvement, IPI risk score, B symptom, tumor size, cell of origin (Hans), and proliferation index were evaluated in our study. These did not display an obvious correlation with early failure of frontline R-CHOP chemo-immunotherapy.

**Figure 1:**
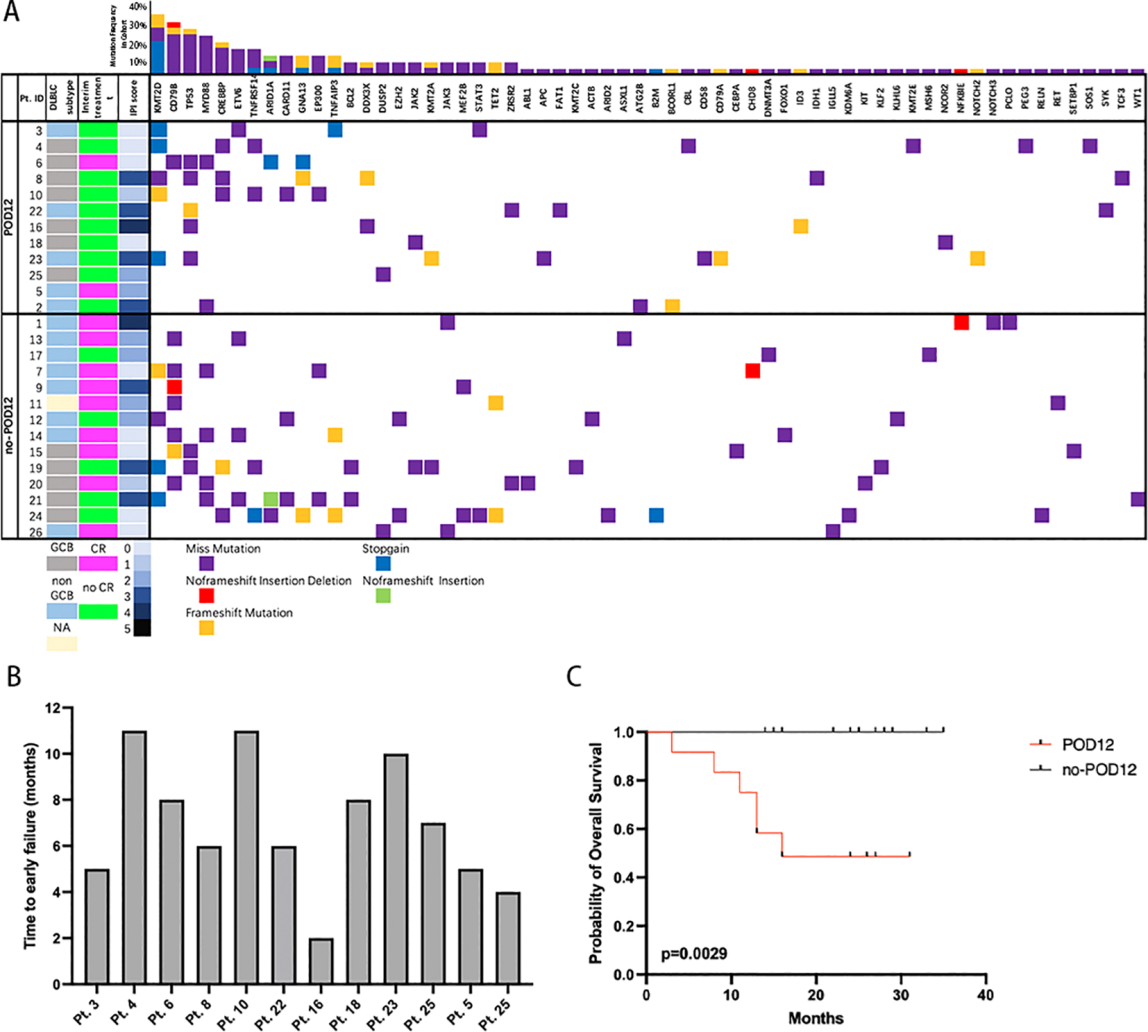
**(A)** Comprehensive mutational profile of newly diagnosed DLBCL cohort. The mutation profile was determined by next-generation sequencing analysis of 103 genes. The mutational spectrum of the patients is grouped according to POD12 or no-POD12. On the top of the mutational heatmap, the bar graph indicates the gene-level alteration type breakdown using the same five-color scheme. The clinical features of 26 patients are on the left side of the mutational heatmap, including the international prognostic index (IPI) score, interim response to therapy (complete response, CR or no CR), germinal center B cell (GCB) versus non-GCB DLBCL subtype. **(B)** Time to early failure from diagnosis in POD12 group. **(C)** The POD12 group had significantly poor overall survival compared with the no-POD12 group. (p=0.0029).

Time to early failure within 12 months from diagnosis in the POD12 group was shown in **Figure 1B**. The median time to progress was 6.5 months. As expected, compared with the no-POD12 group, the POD12 group was related to the poorer OS with a hazard ratio (HR) of 12.13 (95% confidence interval [CI] 2.34–62.78; log-rank test, p=0.0029) (**Figure 1C**). Of the 12 POD12 patients, 6 eventually died during the follow-up, with disease progression being the cause of death in most cases (83.3%).

### Correlation of NGS data to clinical outcomes

**Figure 2:**
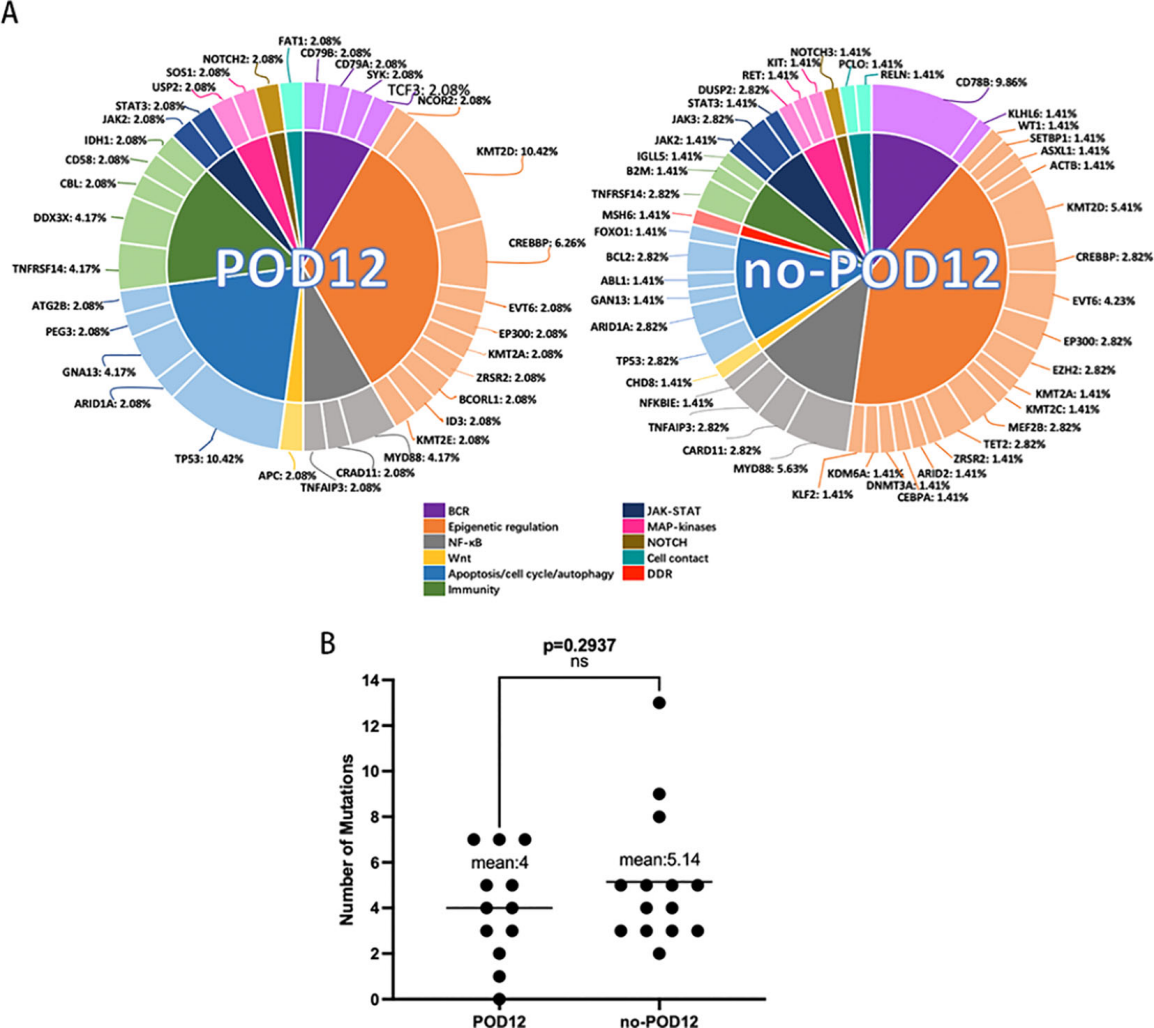
**(A)** Mutation pathway heterogeneity between POD12 and no-POD12 groups. Mutation frequencies per gene are shown here as the percentage of the total number of variants. **(B)** The number of mutations did not correlate with the early failure in DLBCL patients treated with R-CHOP chemo-immunotherapy. (p=0.2937 ns, not statistically significant.

All of these genes were mutated in 96.15% of patients (25/26) with a median number of 4 (0–13), including single nucleotide variants (SNVs) and insertion-deletion events (indels) (**Figure 1A**). Overall, 119 somatic mutations were identified in 26 patients across 69 unique genes, from the 103 targeted gene panels. Mutations that were identified as potential germline variants and mutations with mutant allele frequencies of <5% were filtered from the dataset and excluded in the correlative analyses to clinical outcomes. Of these 119 mutations, the majority were nonsynonymous SNVs and stop gain (n=98, 82.4%). A small number of frameshift mutations (n=17, 14.3%), 1 nonframeshift deletion, and 3 nonframeshift insertion-deletion were identified. The mean number of mutations in POD12 groups was 4 (0-7), and in no-POD12 groups was 5.14 (2-13) (**Figure 2B**), however, no significant difference between the two groups was observed (p=0.2937, t-test). Pt.5 with no detectable mutations corresponded to the POD12 group.

These somatic mutation target genes were grouped into 11 specific pathways: BCR signaling (*CD79B, CD79A, KLHL6, SYK, and TCF3*), epigenetic regulation (*NCOR2, WT1, SETBP1, ASXL1, ACTB, KMT2D, CREBBP, ETV6, EP300, EZH2, KMT2A, KMT2C, MEF2B, TET2, ZRSR2, ARID2, BCORL1, CEBPA, DNMT3A, ID3, KDM6A, KLF2, and KMT2E), NF-kB pathway (MYD88, CARD11, TNFAIP3, and NFKBIE)*, Wnt signaling (*APC* and *CHD8*), apoptosis/cell cycle/autophagy (*TP53, ARID1A, GNA13, ABL1, BCL2, FOXO1, PEG3*, and *ATG2B*), immunity (*TNFRSF14, DDX3X, B2M, CBL, CD58, IDH1*, and *IGLL5*), JACK-STAT (*JAK2, JAK3, and STAT3*), MAP-kinases (*DUSP2, RET, KIT*, and *SOS1*), NOTCH signaling (*NOTCH2* and *NOTCH3*), cell contact (*FAT1, PCLO*, and *RELN*) and DDR (*MSH6*). We then demonstrated that the POD12 subtype was mostly characterized by mutations in the epigenetic modulation pathway (33.32% of total variation) and apoptosis/cell cycle/autophagy pathway (20.83% of total variation) (**Figure 2A**), whereas the no-POD12 subtype is mostly characterized by mutations in the epigenetic modulation pathway (40.66% of total variation) (**Figure 2A**).

Additionally, the risk of POD12 was assessed for the different subtypes of the molecular classification by Wright et al. (11), using the recently described algorithm. The molecular subtypes showed no difference in the POD12 group and the no-POD12 group (data not shown).

### *CD79B* mutations are related to POD12 following R-CHOP treatment

**Figure 3:**
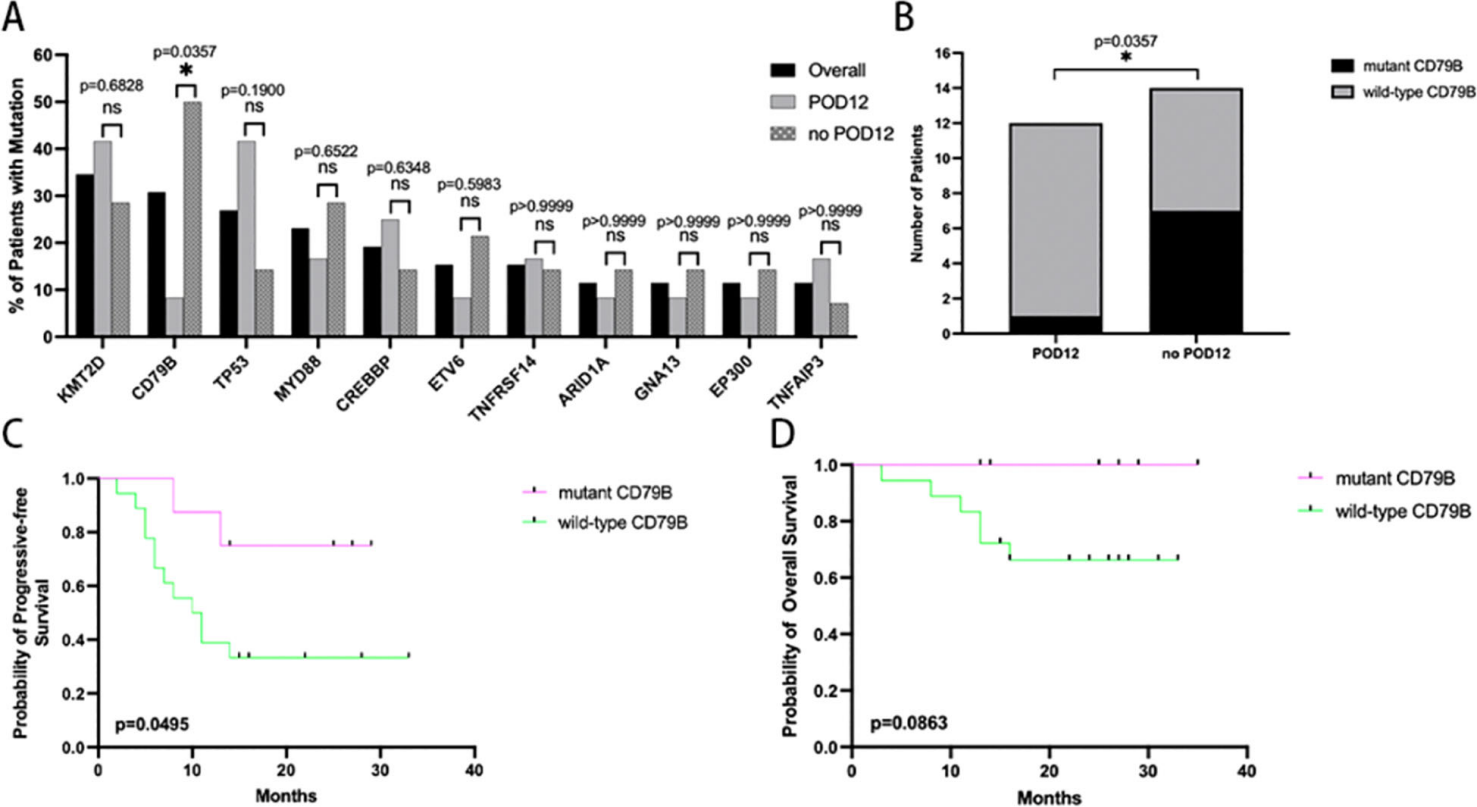
The genetic features of CD79B association with survival. **(A)** Of the high-frequency mutated genes, only CD79B was associated with early failure. **(B)** The no-POD12 group was found to have significantly more mutations in the CD78B gene than the POD12 group. (p=0.0357) **(C)** The wild-type CD79B group had significantly poor progressive-free survival compared with the mutant CD79B group. (p=0.0495) **(D)** However, there was no significant difference in overall survival between the wild-type CD79B group and the mutant CD79B group. (p=0.0863).

The most frequently mutated genes occurring in >10% of all samples included *KMT2D* (34.6%), *CD79B* (30.8%), *TP53* (26.9%), *MYD88* (23.1%), *CREBBP* (19.2%), *ETV6* (15.4%), *TNFRSF14* (15.4%), *CARID1A* (11.5%), *GNA13* (11.5%), *EP300* (11.5%), *TNFAIP3* (11.5%) (**Figure 3A**). Missense mutations comprised of 65.5% (n=38) of all somatic mutations. The others were 9 stop gains, 9 frameshift mutation, 1 no frameshift insertion, and 1 no frameshift insertion deletion. 15 patients (57.7%) had at least two genes concurrent mutations. In this cohort, *CD79B* mutation frequency was significantly increased in the no-POD12 group compared with the POD12 group (50.00% vs 8.33%, P = 0.0357). (**Figure 3B**). By analyzing clinicopathological features, we demonstrated that patients with mutant-type *CD79B* manifested better PFS than wild-type patients (HR of 0.3334 (95% confidence interval [CI] 0.1114–0.9977; log-rank test, p=0.0495), but not OS (HR of 0.2213 (95% confidence interval [CI] 0.0395–1.240; log-rank test, p=0.0883). The 2-year PFS rate were 75.0 and 33.3%, respectively. The 2-year OS rate was 100.0 and 66.2%, respectively (**Figures 3C, D**).

### Interim treatment responses were correlated with POD12 following R-CHOP treatment

**Figure 4:**
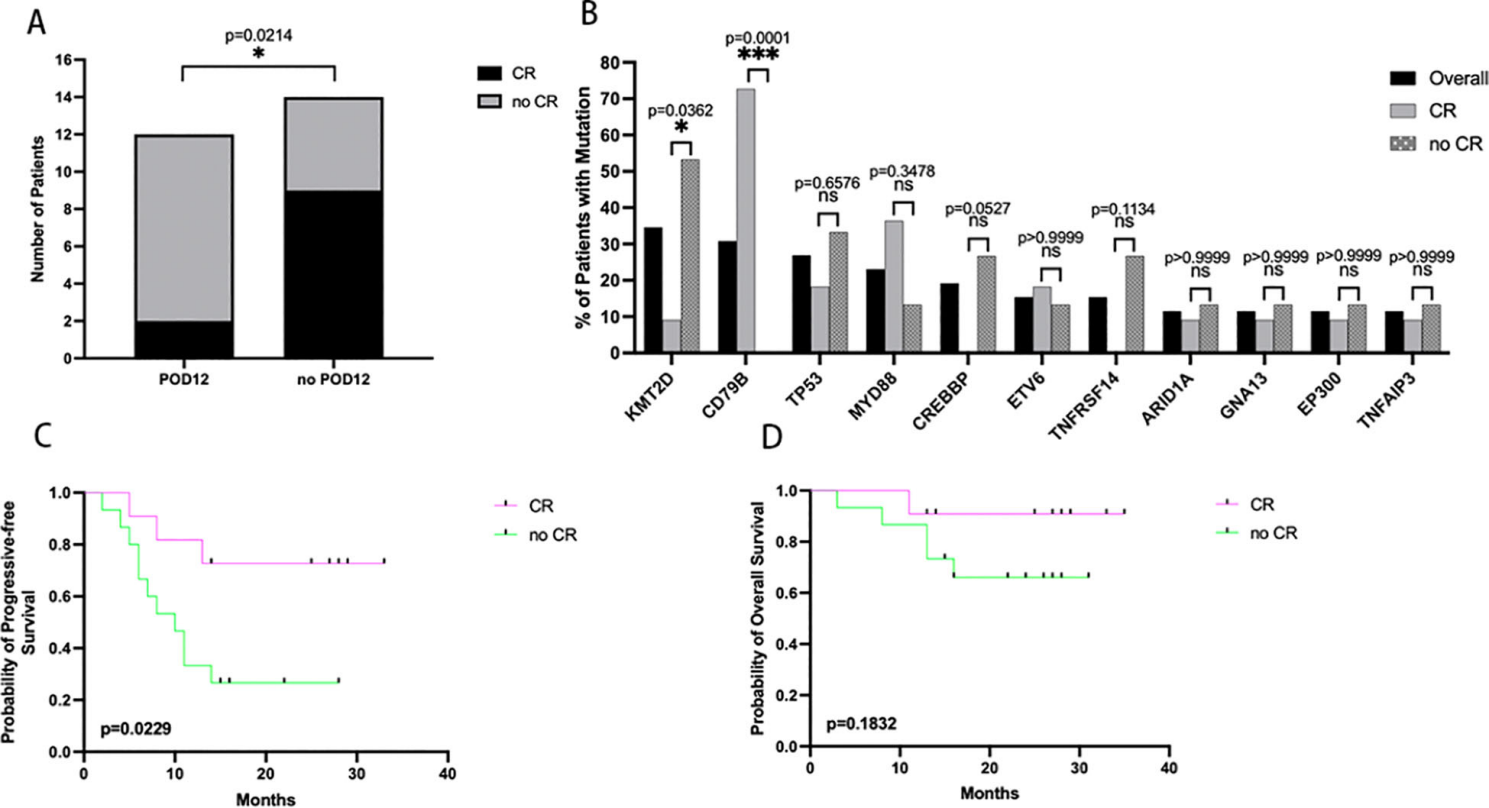
The interim treatment responses are associated with survival. **(A)** The POD12 group was found to have significantly more CR patients than the no-POD12 group. (p=0.0214) **(B)** Of the high-frequency mutated genes, KMT2D (p=0.0362) and CD79B (p=0.0001) were associated with interim treatment responses. **(C)** The no-CR group had significantly poor progressive-free survival compared with the CR group. (p=0.0229) **(D)** However, there was no significant difference in overall survival between the CR group and the no-CR group. (p=0.1832).

Interim treatment responses that did not achieve a complete response (CR) were found to be significantly associated with the POD12 group (**Figure 4A**; p=0.0214, t-test). Two of the 12 patients in the POD12 group (16.67%) had achieved a CR after 4 cycles of R-CHOP chemo-immunotherapy, compared with 64.29% (9 out of 14 patients) in the no-POD12 group. We further demonstrated that patients with CR had better PFS than non-CR patients. The 2-year PFS rates were 72.7 and 26.7%, respectively (HR of 0.2876 (95% confidence interval [CI] 0.0983–0.8414; log-rank test, p=0.0229). The 2-year OS rates were 90.9 and 66.0%, respectively (HR of 0.3311 (95% confidence interval [CI] 0.0650–1.686; log-rank test, p=0.1832). (**Figures 4C, D**), however, there was no significant statistical difference.

We further investigated the correlation between clinical outcomes and genetic mutations. The number of CD79B mutations was significantly increased in the CR group compared with the non-CR group (**Figure 4B**; p=0.0001). However, a higher number of KMT2D mutations were found in the non-CR group compared to the CR group (**Figure 4B**; p=0.0362).

## Discussion

Early failure of frontline treatment is a well-known surrogate marker for biologically more aggressive diseases in hematological malignancies, especially relapse within less than 12 months (POD12), and DLBCL is no exception. In our current analysis, compared with the no-POD12 group, the POD12 group was related to poor OS. The prognostic factors associated with diffuse large B-cell lymphoma (DLBCL) patients receiving R-CHOP chemo-immunotherapy are crucial for identifying the patients at high risk of recurrence and selecting appropriate treatment strategies for them. The International Prognostic Index (IPI) is currently the internationally recognized prognostic indicator (14) and is widely used for risk stratification before treatment, but its prognostic value has been challenged to a certain extent in the era of Rituximab treatment. Our study confirmed the correlation between IPI and early failure in DLBCL patients receiving R-CHOP chemo-immunotherapy, while analysis showed that IPI is not a valid predictor, similar to the results obtained by previous studies (15). Clinical studies have also shown that while IPI can accurately evaluate the prognosis of most DLBCL patients, a part of patients with similar IPI scores still has different rates of long-term survival (14). Therefore, the individual characteristics and chemotherapy response of each patient are considered to be the best indicators of prognosis.

In this study, we observed a panel of 103 genes and a median number of 4 mutations in DLBCL patients, which is similar to the findings in the study containing a larger amount of candidate cancer genes (CCGs) (8). There is speculation that the presence of frequently mutated genes in DLBCL may serve as potential indicators of prognosis. In this cohort, the most mutated genes identified included *KMT2D, CD79B, TP53, MYD88, CREBBP, ETV6, TNFRSF14, CARID1A, GNA13, EP300*, and *TNFAIP3*. However, only *CD79B* gene mutations were found to be significantly different between the POD12 group and the no-POD12 group.

CD79 is a heterodimeric protein consisting of two transmembrane subunits, CD79A, and CD79B, that represent the signal transduction portion of the B-cell receptor (BCR) (16). Hotspot mutations in the BCR CD79B subunit were found in about 30% of ABC-type DLBCL, but only 3% of GCB-type DLBCL (15). These mutations play a central role in BCR activation, enhance “chronic active” BCR signaling by blocking BCR internalization, and can inhibit negative regulators such as LYN and increase b cell surface BCR expression (++ IgM) (17, 18). *CD79B* functional acquisition mutations usually occur simultaneously with *MYD88* mutations and are significantly more frequent in ABC DLBCLs, while *CD79A* mutations are less common, accounting for 2.9% to 4% of cases (17). In this cohort, 8 patients had *CD79B* mutations, one in the POD12 group, the other 7 in the no-POD12 group, and 4 patients had *MYD88* and *CD79* mutations, one in the POD12 group, and the other 3 in the no-POD12 group. The prognostic significance of *CD79B* mutations in DLBCLs is unclear. Although some authors have reported that *CD79B* mutations may lead to survival defects (9). In our study, patients with wild-type *CD79B* manifested poorer PFS than mutant patients, but not OS. Further studies are needed to evaluate the prognostic role of *CD79B* mutations in wild-type *MYD88* with a larger sample size.

Previous studies have shown that interim treatment responses to frontline Rituximab-containing chemo-immunotherapy are an important prognostic factor for DLBCL patients. Patients with poor response to interim treatment and without complete response (CR) were more likely to relapse and progress, and the prognosis was generally worse (19, 20). Our study showed that the risk of POD12 in the no CR patients was significantly higher than that in CR patients, and interim treatment response acted as an independent predictor of 2-year PFS, but not OS.

Only 20 years ago, CHOP in combination with Rituximab (R‐CHOP), the first monoclonal antibody against CD20, significantly changed the outcome of DLBCL patients and became the new standard frontline of treatment (21). Despite these improvements, about a third of patients have relapses (22), and only a few can be cured with aggressive salvage treatments such as high-dose chemotherapy and autologous stem cell transplantation (4). Even R‐CHOP-14 used every 14 days, failed to show survival benefits when compared to standard R‐CHOP-21 (23). The results of our studies suggest that early failure in newly diagnosed DLBCL could be evaluated in the context of particular genetic aberrations and interim treatment responses. This finding provides the basis for novel therapies based primarily on the R‐CHOP backbone. The ultimate goal is to tailor treatment to the biology of every DLBCL patient. For example, drugs that target histone modification (e.g., HDAC inhibitors) could be investigated in whether the response is correlated with mutations in epigenetic regulation signaling. If a patient had mutations in epigenetic regulation and interim treatment response was no-CR, HDAC inhibitors can be considered in addition to R-CHOP, in the chemo-immunotherapy.

## Conclusion

In summary, we reported our center’s experience in genetic and clinical characteristics for early failure of frontline Rituximab-containing chemo-immunotherapy in the newly diagnosed DLBCL cohort. CD79B wild-type and interim treatment responses cannot achieve complete response and are powerfully identified as a subset of DLBCL patients prone to early progression and in need of alternative treatment beyond standard immunochemotherapy in the frontline treatment. Our study may provide some experience on which to develop precision therapies for DLBCL.

## Data Availability

The original contributions presented in the study are included in the article/supplementary material. Further inquiries can be directed to the corresponding authors.

## References

[1] Swerdlow SH Campo E Pileri SA Harris NL Stein H Siebert R . The 2016 revision of the World Health Organization classification of lymphoid neoplasms. Blood. (2016) 127:2375–90. doi: 10.1182/blood-2016-01-643569, PMID: 26980727 PMC4874220

[2] Candelaria M Duenas-Gonzalez A . Rituximab in combination with cyclophosphamide, doxorubicin, vincristine, and prednisone (R-CHOP) in diffuse large B-cell lymphoma. Ther Adv Hematol. (2021) 12:2040620721989579. doi: 10.1177/2040620721989579, PMID: 33796235 PMC7970687

[3] Coiffier B Thieblemont C Van Den Neste E Lepeu G Plantier I Castaigne S . Long-term outcome of patients in the LNH-98.5 trial, the first randomized study comparing rituximab-CHOP to standard CHOP chemotherapy in DLBCL patients: a study by the Groupe d’Etudes des Lymphomes de l’Adulte. Blood. (2010) 116:2040–5. doi: 10.1182/blood-2010-03-276246, PMID: 20548096 PMC2951853

[4] Gisselbrecht C Glass B Mounier N Singh Gill D Linch DC Trneny M . Salvage regimens with autologous transplantation for relapsed large B-cell lymphoma in the rituximab era. J Clin Oncol. (2010) 28:4184–90. doi: 10.1200/JCO.2010.28.1618, PMID: 20660832 PMC3664033

[5] Ruppert AS Dixon JG Salles G Wall A Cunningham D Poeschel V . International prognostic indices in diffuse large B-cell lymphoma: a comparison of IPI, R-IPI, and NCCN-IPI. Blood. (2020) 135:2041–8. doi: 10.1182/blood.2019002729, PMID: 32232482

[6] Morin RD Mungall K Pleasance E Mungall AJ Goya R Huff RD . Mutational and structural analysis of diffuse large B-cell lymphoma using whole-genome sequencing. Blood. (2013) 122:1256–65. doi: 10.1182/blood-2013-02-483727, PMID: 23699601 PMC3744992

[7] Zhang J Grubor V Love CL Banerjee A Richards KL Mieczkowski PA . Genetic heterogeneity of diffuse large B-cell lymphoma. Proc Natl Acad Sci U S A. (2013) 110:1398–403. doi: 10.1073/pnas.1205299110, PMID: 23292937 PMC3557051

[8] Chapuy B Stewart C Dunford AJ Kim J Kamburov A Redd RA . Molecular subtypes of diffuse large B cell lymphoma are associated with distinct pathogenic mechanisms and outcomes. Nat Med. (2018) 24:679–90. doi: 10.1038/s41591-018-0016-8, PMID: 29713087 PMC6613387

[9] Reddy A Zhang J Davis NS Moffitt AB Love CL Waldrop A . Genetic and functional drivers of diffuse large B cell lymphoma. Cell. (2017) 171:481–94 e15. doi: 10.1016/j.cell.2017.09.027, PMID: 28985567 PMC5659841

[10] Schmitz R Wright GW Huang DW Johnson CA Phelan JD Wang JQ . Genetics and pathogenesis of diffuse large B-cell lymphoma. N Engl J Med. (2018) 378:1396–407. doi: 10.1056/NEJMoa1801445, PMID: 29641966 PMC6010183

[11] Wright GW Huang DW Phelan JD Coulibaly ZA Roulland S Young RM . A probabilistic classification tool for genetic subtypes of diffuse large B cell lymphoma with therapeutic implications. Cancer Cell. (2020) 37:551–68 e14. doi: 10.1016/j.ccell.2020.03.015, PMID: 32289277 PMC8459709

[12] Dubois S Viailly PJ Mareschal S Bohers E Bertrand P Ruminy P . Next-generation sequencing in diffuse large B-cell lymphoma highlights molecular divergence and therapeutic opportunities: a LYSA study. Clin Cancer Res. (2016) 22:2919–28. doi: 10.1158/1078-0432.CCR-15-2305, PMID: 26819451

[13] Cheson BD Fisher RI Barrington SF Cavalli F Schwartz LH Zucca E . Recommendations for initial evaluation, staging, and response assessment of Hodgkin and non-Hodgkin lymphoma: the Lugano classification. J Clin Oncol. (2014) 32:3059–68. doi: 10.1200/JCO.2013.54.8800, PMID: 25113753 PMC4979083

[14] Sehn LH Berry B Chhanabhai M Fitzgerald C Gill K Hoskins P . The revised International Prognostic Index (R-IPI) is a better predictor of outcome than the standard IPI for patients with diffuse large B-cell lymphoma treated with R-CHOP. Blood. (2007) 109:1857–61. doi: 10.1182/blood-2006-08-038257, PMID: 17105812

[15] Kwon SH Kang DR Kim J Yoon JK Lee SJ Jeong SH . Prognostic value of negative interim 2-[^18^F]-fluoro-2-deoxy-d-glucose PET/CT in diffuse large B-cell lymphoma. Clin Radiol. (2016) 71:280–6. doi: 10.1016/j.crad.2015.11.019, PMID: 26732889

[16] Visco C Tanasi I Quaglia FM Ferrarini I Fraenza C Krampera M . Oncogenic mutations of MYD88 and CD79B in diffuse large B-cell lymphoma and implications for clinical practice. Cancers (Basel). (2020) 12:2913. doi: 10.3390/cancers12102913, PMID: 33050534 PMC7600909

[17] Davis RE Ngo VN Lenz G Tolar P Young RM Romesser PB . Chronic active B-cell-receptor signalling in diffuse large B-cell lymphoma. Nature. (2010) 463:88–92. doi: 10.1038/nature08638, PMID: 20054396 PMC2845535

[18] Dal Porto JM Gauld SB Merrell KT Mills D Pugh-Bernard AE Cambier J . B cell antigen receptor signaling 101. Mol Immunol. (2004) 41:599–613. doi: 10.1016/j.molimm.2004.04.008, PMID: 15219998

[19] Zhu L Meng Y Guo L Zhao H Shi Y Li S . Predictive value of baseline (18)F-FDG PET/CT and interim treatment response for the prognosis of patients with diffuse large B-cell lymphoma receiving R-CHOP chemotherapy. Oncol Lett. (2021) 21:132. doi: 10.3892/ol.2020.12393, PMID: 33552253 PMC7798034

[20] Oñate-Ocaña LF Cortés V Castillo-Llanos R Terrazas A Garcia-Perez O Pitalúa-Cortes Q . Metabolic tumor volume changes assessed by interval (18)fluorodeoxyglucose positron emission tomography-computed tomography for the prediction of complete response and survival in patients with diffuse large B-cell lymphoma. Oncol Lett. (2018) 16:1411–8. doi: 10.3892/ol.2018.8817, PMID: 30008818 PMC6036479

[21] Coiffier B Lepage E Briere J Herbrecht R Tilly H Bouabdallah R . CHOP chemotherapy plus rituximab compared with CHOP alone in elderly patients with diffuse large-B-cell lymphoma. N Engl J Med. (2002) 346:235–42. doi: 10.1056/NEJMoa011795, PMID: 11807147

[22] Pfreundschuh M Kuhnt E Trümper L Osterborg A Trneny M Shepherd L . CHOP-like chemotherapy with or without rituximab in young patients with good-prognosis diffuse large-B-cell lymphoma: 6-year results of an open-label randomised study of the MabThera International Trial (MInT) Group. Lancet Oncol. (2011) 12:1013–22. doi: 10.1016/S1470-2045(11)70235-2, PMID: 21940214

[23] Cunningham D Hawkes EA Jack A Qian W Smith P Mouncey P . Rituximab plus cyclophosphamide, doxorubicin, vincristine, and prednisolone in patients with newly diagnosed diffuse large B-cell non-Hodgkin lymphoma: a phase 3 comparison of dose intensification with 14-day versus 21-day cycles. Lancet. (2013) 381:1817–26. doi: 10.1016/S0140-6736(13)60313-X, PMID: 23615461

